# Timing based clustering of childhood BMI trajectories reveals differential maturational patterns; Study in the Northern Finland Birth Cohorts 1966 and 1986

**DOI:** 10.1038/s41366-025-01714-8

**Published:** 2025-01-16

**Authors:** Anni Heiskala, J. Derek Tucker, Priyanka Choudhary, Rozenn Nedelec, Justiina Ronkainen, Olli Sarala, Marjo-Riitta Järvelin, Mikko J. Sillanpää, Sylvain Sebert

**Affiliations:** 1 Research Unit of Population Health, University of Oulu, Oulu, Finland; 2 Statistical Sciences, Sandia National Laboratories, Albuquerque, NM USA; 3 Research Unit of Mathematical Sciences, University of Oulu, Oulu, Finland; 4 MRC Centre for Environment and Health, Department of Epidemiology and Biostatistics, School of Public Health, Imperial College London, London, UK; 5 Department of Life Sciences, College of Health and Life Sciences, Brunel University London, London, UK

**Keywords:** Epidemiology, Risk factors

## Abstract

**Background/Objectives:**

Children’s biological age does not always correspond to their chronological age. In the case of BMI trajectories, this can appear as phase variation, which can be seen as shift, stretch, or shrinking between trajectories. With maturation thought of as a process moving towards the final state - adult BMI, we assessed whether children can be divided into latent groups reflecting similar maturational age of BMI. The groups were characterised by early factors and time-related features of the trajectories.

**Subjects/Methods:**

We used data from two general population birth cohort studies, Northern Finland Birth Cohorts 1966 and 1986 (NFBC1966 and NFBC1986). Height (*n* = 6329) and weight (*n* = 6568) measurements were interpolated in 34 shared time points using B-splines, and BMI values were calculated between 3 months to 16 years. Pairwise phase distances of 2999 females and 3163 males were used as a similarity measure in k-medoids clustering.

**Results:**

We identified three clusters of trajectories in females and males (*Type 1*: females, n = 1566, males, *n* = 1669; *Type 2*: females, *n* = 1028, males, *n* = 973; *Type 3*: females, *n* = 405, males, *n* = 521). Similar distinct timing patterns were identified in males and females. The clusters did not differ by sex, or early growth determinants studied.

**Conclusions:**

Trajectory cluster *Type 1* reflected to the shape of what is typically illustrated as the childhood BMI trajectory in literature. However, the other two have not been identified previously. *Type 2* pattern was more common in the NFBC1966 suggesting a generational shift in BMI maturational patterns.

## Introduction

When studying trajectories of a longitudinal phenomenon in a population, one often observes temporal variation between the individual trajectories. Indeed, although the trajectories exhibit similar shapes, they might seem slightly shifted, stretched, or shrunk in relation to each other. This is called phase variation in functional data analysis [1]. Phase variation related to the description of biological phenotypes such as human growth has received attention in the past, with height [2, 3] and leg growth [4]. More recently also multivariate functional data have been considered, with some examples related to growth [5, 6]. To date, the approaches to model longitudinal changes of BMI in children have not considered phase variation.

Previous studies have identified common inflection points in the childhood BMI trajectory, namely the infancy peak [7] and adiposity rebound point (AR) [8]. However, substantial variation is reported in the timing of these inflection points across populations [9, 10] with average age at infancy peak ranging between 6.0 and 9.9 months [11, 12], and average age at AR between 3.0 and 8.7 years [13, 14]. This suggests that the biological meaning of childhood BMI may differ between children of the same age with different growth patterns in bone, muscle, and adipose tissues [13]. Furthermore, in a British birth cohort study, over 30% of adolescents that were categorised to be with overweight or obesity according to sex and age adjusted BMI cut-offs, were reclassified to a lower weight BMI category when biological maturation was controlled for [15]. Therefore, it might be too ambiguous to refer only to a chronological age when describing children’s cross-sectional BMI.

Childhood height growth can be thought of as a process of maturation towards the mature state, e.g., adult height [16]. The same analogy could be used for BMI, if we assume that adult BMI is a stable phenotype that occurs after the pubertal processes have ended. When assuming this, we must distinguish between the *chronological age* and *maturational age*. Two children are at the same maturational age regarding childhood BMI when they are in the same phase of their BMI development, regardless of their chronological age. More generally, the different time scales are sometimes referred to as clock time and system time [1].

In the present study, we hypothesised that there are latent groups with distinct childhood BMI timing patterns. To address this, we (1) assessed whether children can be divided into latent groups that reflect similar maturational age regarding BMI from 3 months to 16 years, and (2) characterised the groups by early factors and time-related features of the trajectories. The difference in maturational age was measured by phase distances of the BMI trajectories, which quantify how well two curves are aligned with one another. Clustering by phase distances without aligning does not remove the phase variation but allows to identify latent groups where the phase distances are more alike, i.e. the BMI trajectories of the children are similar in respect to timing of BMI maturation. It may also help to identify different shape patterns of childhood BMI if such patterns exist in the data.

## Material and methods

### Study participants

We used data from two longitudinal birth cohorts born in Northern Finland twenty years apart.

#### Northern Finland Birth Cohort 1966

Northern Finland Birth Cohort 1966 (NFBC1966) [17] is a birth cohort of pregnant women and their offspring from the Finnish provinces of Oulu and Lapland. The NFBC1966 targeted all expected deliveries during a one-year period 1st of January 1966 to 31st of December 1966. It comprised 96,3% of all deliveries in the area for this time period, consisting of 12,055 mothers and 12,058 live born offsprings [18]. The mothers were followed up in antenatal clinics by mid-wives and completed three questionnaires in different phases of their pregnancy, complemented with information on the childbirth. Information on the offspring has since been gathered through postal questionnaires, clinical examinations, and health records.

#### Northern Finland Birth Cohort 1986

The Northern Finland Birth Cohort 1986 (NFBC1986) [19] was initiated 20 years after the NFBC1966 in the same two northernmost provinces of Finland. It included 99% of all deliveries in the area, with a due date between 1st of July 1985 and 30th of June 1986 [20, 21]. This corresponded to 9362 mothers and 9432 live births. The mothers were followed up similar to NFBC1966 described above and filled in questionnaires three times during the pregnancy. Information on the offsprings has since been complemented through postal questionnaires, clinical examinations, and health records.

### Height and weight measurements

Information on height (cm) and weight (kg), was obtained by healthcare professionals and linked to the data; first, from primary healthcare records (regular health check-ups from birth until school age, i.e. 7 years) and school health records afterwards. In addition, the participants of the NFBC1986 were invited to a clinical examination at the age of 16 years where height and weight were measured by research nurses. Up to 43 weight observations were available for 7865 participants in the NFBC1966 and up to 58 observations of 6942 participants in the NFBC1986. For height, at most 42 observations were available for 7852 participants in the NFBC1966 and 51 observations for 6941 participants in the NFBC1986.

### Selection criteria of height and weight measurements

Subsequent analysis required common BMI timepoints between individuals, and therefore, we applied spline interpolation for measured values of height and weight. To provide dense height and weight curves for interpolation, we defined the selection criteria as follows:

1. Earliest measurement taken before 6 months of age (excluding birth measures).
2. Last measurement taken after 16 years.
3. Intervals between subsequent measurements less than 5 years.

### Demographic variables

For characterisation of the study sample, the following maternal variables were reported: age at delivery, cigarette smoking during pregnancy, education level, pre-pregnancy BMI, parity, and place of residence. For the participants, the following variables were reported: mode of delivery, single or multiple birth, gestational age, prematurity, and birth weight. Details of how the demographic variables were classified are available in the Supplementary Text S1.1.

### Statistical analyses

#### Pre-processing height and weight measurements

Pre-processing of height and weight measurements is described in detail in Supplementary Text S1.2. In brief, height and weight were interpolated in one cohort at a time using B-splines to derive BMI estimates at desired timepoints. In all trajectories, knot points were placed at the individual’s minimum and maximum ages as well as at 3, 12, 48, 96 and 144 months. Degrees of best fitting splines within cohort were determined by visually inspecting randomly selected individual’s curves, as well as by checking decreases of 1 cm in height or 2 kg in weight within the trajectory. In the final fits, we assessed all decreasing trajectories again, and excluded the participant from further analysis where the spline fit seemed to produce anomalies in the trajectory that were not observed from the height or weight measurements. B-spline interpolation was carried out using the R package fda [22]. R script for the analysis is provided in Supplementary script.

BMI estimates were then calculated as weight(kg)/height(m)^2^ from the extracted height and weight measurements. A total of 34 data points of 6162 individuals were analysed with time points at 3, 6, 9 and 12 months, and then every 6 months up to 16 years of age.

As a sensitivity analysis, to assess the stability of results regarding the interpolation, we used linear interpolation [23] for pre-processing height and weight measurements. The description of the procedure is presented in the Supplementary Text S1.3.

#### Similarity distances

For estimating similarity distances, the two cohorts were pooled and stratified by sex. Pooling was done as the preliminary cohort specific analyses revealed similar clusters in both NFBC1966 and NFBC1986.

Pairwise elastic phase distances [24] were computed between individuals in sex-specific datasets. In general terms, phase distance measures how well two functions are aligned with one another. In this context, the BMI trajectories are thought of as functions of time. Separating pairwise amplitude and phase distances between functions ${f}_{1}$ and ${f}_{2}$ is based on aligning the two functions. Technically, aligning ${f}_{2}$ to ${f}_{1}$ means finding a warping function ${\rm{\gamma }}$ such that the composition ${f}_{2}\circ {\rm{\gamma }}$ is most similar to ${f}_{1}$. The phase distance between functions ${f}_{1}$ and ${f}_{2}$ is defined as the arc-length between the square-root slope function (SRSF) of ${f}_{1}$ and the SRSF of the warping function *γ* on a unit sphere. For more details and full definition of the SRSF, refer to previous works from Tucker et al. and Srivastava et al. [24, 25].

Phase distances were calculated using the R function elastic.distance in the package fdasrvf [26].

#### Clustering

Phase distances were used in the dissimilarity matrix in partitioning around medoids approach (PAM or k-medoids) with the number of clusters *k* set between 2 and 10. Average silhouette widths were used to determine the optimal value for *k*. The average silhouette width is a popular metric for cluster quality, suggested to be used with PAM [27]. Clustering was done with the function pam in the R package cluster [28]. A general description of the steps to perform phase clustering from repeated height and weight measures is illustrated in the Supplementary Fig. 1. R script for the corresponding process is given in Supplementary Script.

#### Characteristics of the clusters

Of the previously mentioned demographic variables, we reported means along with standard deviations for continuous variables, and number of participants with the percentage of the total sample for categorical variables in each of the identified clusters. Cluster-specific medians as well as 1st and 3rd quartiles of BMI trajectories were further reported regarding the following timing-related measures: age at reaching the first peak (i.e., infancy peak), age at reaching the first valley (i.e., adiposity rebound point), the average rate of change in BMI between the beginning of the estimated trajectory at three months and the first peak, average rate of change in BMI between the first peak and the next valley (i.e., between infancy peak and adiposity rebound point) as well as the duration between the first peak and the first valley (i.e., from infancy peak to adiposity rebound point).

To assess the cluster-specific average amplitude or level of BMI, we performed additional analyses where trajectories were aligned to their cluster centroid. Further reasoning, as well as the detailed description of methods, is presented in the Supplementary Text S1.4.

All analyses were conducted in R [29] versions 4.2.3 and 4.3.1.

## Results

The number of participants included after each of the selection criteria step in this study can be found in the flowchart Supplementary Fig. 2. More details regarding attrition are available in Supplementary Text S2.1 and Supplementary Table 1.

**Fig. 1:**
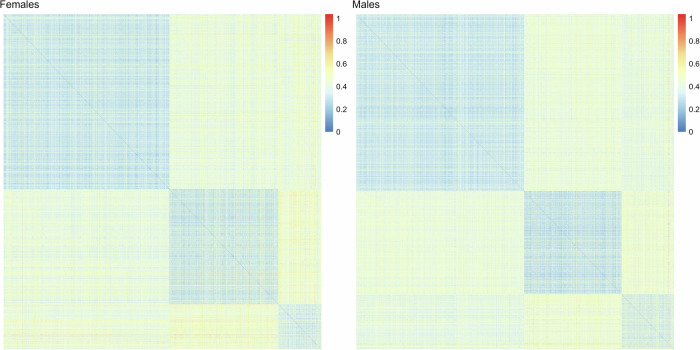
Cluster-wise ordered heatmaps of pairwise phase distances in females on the left and males on the right. Each row and column represent an individual. Each intersection point displays the phase distance between the trajectories of the corresponding individuals as a colour. The three BMI trajectory clusters appear as darker blocks, with blue indicating distance close to zero and yellow and red colours indicating greater distance.

In cluster analysis of females, the maximum average silhouette width was found with the number of clusters *k* = *3 (average silhouette width* = *0.20)*. In males, the maximum average silhouette width was found when *k* = *2 (0.21)*, and a similar average silhouette width with *k* = *3 (0.20)*. Number of cluster *k* = *3* was chosen for both sexes for comparability of clusters. Supplementary Table 2 provides details of average silhouette widths with different number of clusters *k*. Cluster-wise ordered heatmaps of phase distances showed good clusterability in both sexes (Fig. 1). The identified clusters were named as *“Type 1”* (n_females_=1566; n_males_ = 1669), *“Type 2”* (n_females_=1028; n_males_ = 973) and *“Type 3”* (n_females_=405; n_males_ = 521), according to the decreasing order of cluster sizes (Table 1).

**Table 1:** Cluster sizes and proportions for females and males.

Cluster type	Females *n* (%)	Males *n* (%)
Type 1	1566 (52.2)	1669 (52.8)
Type 2	1028 (34.3)	973 (30.8)
Type 3	405 (13.5)	521 (16.5)
Total	2999 (100)	3163 (100)

Individuals from the two cohorts were not evenly distributed in the discovered clusters (Table 2). *Type 1* in both sexes and *Type 3* in females had more participants from the NFBC1986, and *Type 2* in both sexes as well as *Type 3* in males were more often from the NFBC1966. *Type 3* participants’ mothers had more often continued smoking after the 2nd month of the pregnancy and had lower educational level than mothers of the participants clustered to *Type 1* or *Type 2*. Although the sample size for multiple births was relatively low, *Type 2* individuals were less likely to be twins.

**Table 2:** Characteristics of the studied population per BMI trajectory clusters. With continuous variables, mean (standard deviation, SD) and with categorical variables, *n* (%) reported.

	Female BMI trajectory types			Male BMI trajectory types		
Variable /levels	Type 1	Type 2	Type 3	Type 1	Type 2	Type 3
**Cohort**						
NFBC1966	663 (42.3)	597 (58.1)	193 (47.7)	678 (40.6)	645 (66.3)	288 (55.3)
NFBC1986	903 (57.7)	431 (41.9)	212 (52.3)	991 (59.4)	328 (33.7)	233 (44.7)
**Average number of height measurements**	22.8 (4.4)	22.7 (4.8)	22.7 (5.0)	22.7 (4.3)	22.5 (4.8)	21.9 (4.5)
**Average number of weight measurements**	24.3 (4.9)	24.0 (5.4)	24.4 (5.8)	24.2 (4.8)	23.5 (5.1)	23.2 (5.0)
**Maternal age at birth, years**	27.7 (5.8)	27.5 (5.8)	28.0 (6.3)	28.0 (5.7)	27.7 (5.9)	27.9 (5.8)
**Maternal smoking during pregnancy**						
No	1210 (78.0)	841 (82.7)	300 (75.0)	1303 (79.0)	759 (79.6)	397 (76.9)
Yes, quitted	79 (5.1)	41 (4.0)	21 (5.2)	73 (4.4)	62 (6.5)	26 (5.0)
Yes	262 (16.9)	135 (13.3)	79 (19.8)	274 (16.6)	133 (13.9)	93 (18.0)
**Maternal education**						
High	250 (17.0)	149 (15.2)	54 (14.0)	232 (15.0)	131 (14.0)	60 (12.0)
Medium	411 (27.9)	262 (26.8)	96 (24.8)	473 (30.5)	230 (24.7)	131 (27.5)
Low	811 (55.1)	568 (58.0)	237 (61.2)	845 (54.5)	572 (61.3)	286 (60.0)
**Maternal pre-pregnancy BMI, kg/m**^**2**^	22.7 (3.3)	22.3 (2.9)	23.2 (3.7)	22.7 (3.4)	22.4 (3.0)	22.6 (3.0)
**Mode of delivery**						
Non-instrumental vaginal delivery	889 (77.3)	526 (80.2)	237 (82.0)	970 (76.4)	445 (78.3)	274 (79.0)
C-section	170 (14.8)	87 (13.3)	37 (12.8)	170 (13.4)	68 (12.0)	45 (13.0)
Other (vacuum extraction, forceps)	90 (7.9)	43 (6.6)	15 (5.2)	130 (10.2)	55 (9.7)	28 (8.1)
**Multiple birth**						
Singleton	1535 (98.0)	1016 (98.8)	395 (97.5)	1618 (96.9)	956 (98.3)	510 (97.9)
Twin	31 (2.0)	12 (1.2)	10 (2.5)	50 (3.0)	16 (1.6)	11 (2.1)
Triplet	0 (0.0)	0 (0.0)	0 (0.0)	1 (0.1)	1 (0.1)	0 (0.0)
**Parity**						
No	542 (34.7)	360 (35.2)	151 (37.4)	580 (34.8)	353 (36.4)	203 (39.2)
Yes	1022 (65.3)	664 (64.8)	253 (62.6)	1087 (65.2)	618 (63.6)	315 (60.8)
**Gestational age, weeks**	39.9 (1.7)	40.0 (1.7)	40.0 (1.7)	39.8 (1.8)	40.0 (1.6)	39.9 (1.8)
**Birth status**						
Term ( ≥ 37 weeks)	1474 (95.6)	979 (96.5)	384 (95.8)	1544 (93.7)	925 (97.2)	493 (96.7)
Preterm ( < 37 weeks)	68 (4.4)	36 (3.5)	17 (4.2)	104 (6.3)	27 (2.8)	17 (3.3)
**Birth weight, grams**	3455 (500)	3457 (482)	3522 (537)	3573 (547)	3562 (522)	3594 (522)
**Place of residence at birth**						
Town	685 (43.8)	445 (43.3)	185 (45.9)	684 (41.1)	462 (47.5)	209 (40.4)
Village centre	502 (32.1)	331 (32.2)	119 (29.5)	588 (35.3)	297 (30.6)	184 (35.6)
Remote village	378 (24.2)	251 (24.4)	99 (24.6)	392 (23.6)	213 (21.9)	124 (24.0)

**Fig. 2:**
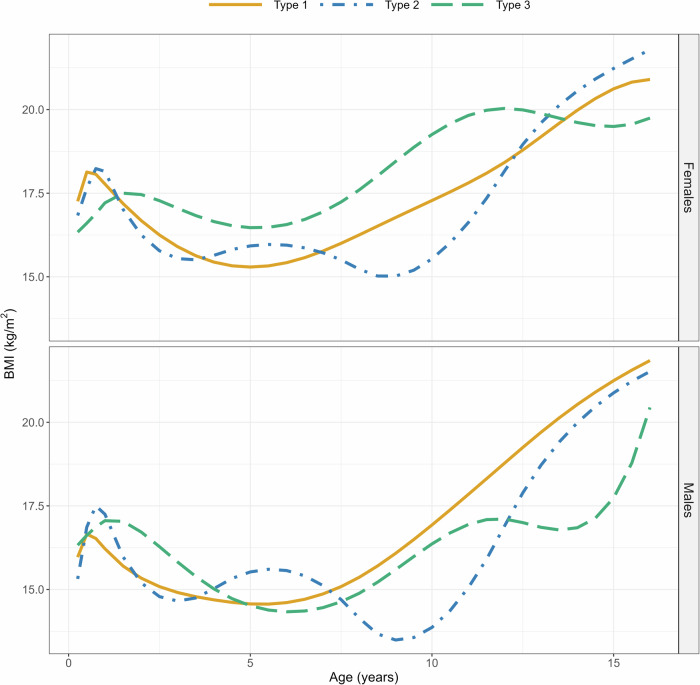
Medoid BMI trajectories of each cluster in females above and males below. A medoid is a representative trajectory from the cluster, where the sum of pairwise phase distances between it and other trajectories within the cluster are at a minimum. In females, Type 1 includes 52.2% of the participants, Type 2 includes 34.3%, and Type 3 includes 13.5%. In males, Type 1 includes 53.8% of the participants, Type 2 includes 30.8%, and Type 3 includes 16.5%.

Females and males had comparable shapes in the cluster centroids (Fig. 2) that seem to reflect the typical BMI trajectory shapes within their clusters (Supplementary Fig. 3). Participants within cluster *Type 1* exhibited a shape with a clear peak observed soon after birth, followed by a decrease until the adiposity rebound point and then increasing again towards its final level. Trajectories of the cluster *Type 2* were characterised by an early peak similar to *Type 1*, but they differed from the other clusters with an intermediate rise in the BMI between the ages of four and nine years.

The time-related BMI trajectory characteristics of the cluster types are shown in Table 3. Median age at 1st peak of the trajectory was at 0.75 years in all clusters and both sexes. Median infancy slopes were steepest in *Type 2* females and males, indicating that individuals in this cluster had highest average rate of change in BMI between the earliest point included in the trajectories (3 months) and the first peak. Median childhood slopes from first peak to the next valley were also the steepest in trajectories of the cluster *Type 2*. The duration from which childhood slope was measured was shortest in *Type 2* and longest in *Type 1* trajectories.

**Table 3:** Main time-related features of the BMI trajectory clusters. Median (1st–3rd quartile) and *n* reported per cluster.

		Age at 1st peak^a^, years		Infancy slope^b^, kg/m^2^years		Age at 1st valley^c^, years		Childhood slope^d^, kg/m^2^years		Duration of BMI decrease in childhood^f^, years	
Sex	Cluster	Median (Q1–Q3)	*n*	Median (Q1–Q3)	*n*	Median (Q1–Q3)	*n*	Median (Q1–Q3)	*n*	Median (Q1–Q3)	*n*
Females	Type 1	0.75 (0.75–1.00)	1496	2.31 (1.22–3.83)	1505	5.00 (4.00–6.50)	1561	−0.55 (−0.78 to −0.39)	1497	4.00 (3.25–5.50)	1497
	Type 2	0.75 (0.75– 1.00)	1017	3.33 (1.78–5.34)	1019	3.00 (2.50–4.50)	1027	−0.82 (-1.21 to −0.51)	1017	2.50 (1.75–3.50)	1017
	Type 3	0.75 (0.75–1.00)	394	2.56 (1.30–4.16)	401	4.00 (2.50–5.50)	401	−0.63 (−0.91 to −0.41)	397	3.50 (2.25–4.50)	397
Males	Type 1	0.75 (0.75–1.00)	1595	2.25 (1.15–3.79)	1606	5.00 (4.00–6.50)	1668	−0.59 (−0.80 to −0.42)	1596	4.25 (3.50–5.75)	1596
	Type 2	0.75 (0.75–0.75)	963	3.22 (1.48–5.05)	966	3.00 (2.50–4.00)	973	−0.95 (-1.38 to −0.62)	964	2.25 (1.75–3.06)	964
	Type 3	0.75 (0.75–1.00)	499	2.43 (1.15–4.14)	514	5.00 (3.50–6.50)	520	−0.64 (−0.82 to −0.47)	513	4.25 (3.00–5.50)	513

^a ”^infancy peak”; ^b^ from 3 months to 1st peak; ^c^ ”adiposity rebound point”; ^d^ from 1st peak to 1st valley; ^f^ duration from 1st peak to 1st valley

In the vast majority ( > 70% in females and males) of trajectories in *Type 1* the number of peak features was one (Table 4). The number of peak features in *Type 2* trajectories was most often two (61% in females and 65% in males) or three (22% in both sexes). Up to 81% of females’ and 71% of males’ *Type 3* trajectories had in total two BMI peaks (Table 4).

**Table 4:** Number (%) of peak features within trajectories in BMI phase clusters in females and males.

	Females			Males		
Number of peaks	Type 1	Type 2	Type 3	Type 1	Type 2	Type 3
Nill	59 (3.8)	9 (0.9)	2 (0.5)	63 (3.8)	7 (0.7)	6 (1.2)
One	1151 (73.5)	164 (16.0)	34 (8.4)	1298 (77.8)	122 (12.5)	110 (21.1)
Two	345 (22.0)	631 (61.4)	328 (81.0)	300 (18.0)	628 (64.5)	370 (71.0)
Three	11 (0.7)	224 (21.8)	41 (10.1)	8 (0.5)	216 (22.2)	35 (6.7)

Cross-sectional average BMI trajectories accompanied by their 95% confidence intervals of aligned BMI trajectories are illustrated in Supplementary Fig. 4. The mean trajectories cross several times, indicating that having a higher average BMI than another cluster at one time point does not mean that the average BMI level is constantly higher than in another cluster.

Clustering results of the sensitivity analysis using linear interpolation for height and weight curves agreed with the main analysis using B-spline interpolation (Supplementary Text S2.2). The overall cluster agreement was 58,5% between the main (using B-splines for height and weight) and sensitivity analysis (using linear interpolation for height and weight). More details of the cluster agreement are presented in Supplementary Table 4. The sensitivity analysis resulted visually similar clusters to the main analysis (Supplementary Figs. 5, 6 and 7).

## Discussion

In the present study, we applied a new method to identify clusters of childhood BMI trajectories based on the phase differences and using repeated measures of height and weight from just after birth until 16 years. We discriminated three clusters of children, each exhibiting similar trajectories in both sexes. The trajectory cluster *Type 1* was similar in shape to the commonly described BMI trajectory [8, 30]. However, to our knowledge, the two additional types of trajectories we observed have not been reported in the literature.

Understanding changes in the trajectory of BMI during childhood has important biological and clinical implications. This should contribute to better understand children’s growth during childhood and to identify individuals deviating from the “healthy reference” by any condition or health-related factor. So far, the focus has mainly been on BMI level or studying critical periods that are known to associate with later BMI or health status.

Clustering based on the timing of the BMI trajectory is fundamentally different to the more traditional efforts to cluster BMI trajectories, as typically, the focus has been on identifying groups according to the level of BMI. This includes assessing whether BMI is stable or unstable over time, and whether it is high or low compared to the average in the population [31–34]. These are relevant questions for better understanding the epidemic of obesity, as it is shown that high BMI in childhood often persists into adulthood [35, 36]. They do not, however, shed light into our interest of timing of BMI maturation.

In the demographic characterisation of clusters, we did not find differences in early factors that could explain where the growth differences, seen as patterns in BMI trajectories, could arise from. However, we observed that the clusters were not evenly distributed between the two cohorts. This is an interesting finding as the cohorts are from the same area in Finland but 20 years apart. Individuals of the NFBC1966 were born to an industrialising agrarian society whereas the NFBC1986 study participants were born and grew up in a much more modern environment than their counterparts two decades earlier. Several factors influencing maternal and child health and care (and growth of the child) advanced in between, including primary care procedures, social security and support, as well as average quality of nutrition. For instance, perinatal mortality decreased from 2.4% in NFBC166 to 0.9% in NFBC1986, and mothers of NFBC1986 were eligible for paid maternity leave four weeks before the estimated birth date [37]. Nedelec et al. have also discussed breastfeeding and how it was promoted by healthcare personnel in Finland between the decades covering the early stages of the two birth cohorts also used in this study [32].

Time-related characteristics (age at first peak, infancy slope, age at first valley, childhood slope and duration of BMI decrease in childhood) are subject to the chosen time points, as the trajectories are represented by the estimated BMI values in the 34 time points. For example, each cluster type has median age at the 1st peak in the BMI at 0.75 years of age with almost identical 1st and 3rd quartile values. It is likely there would be more variation in the ages if we had more than four time points (at 3, 6, 9, and 12 months) selected for the first year. Previous studies have found differences in the age at adiposity peak between NFBC1966 and NFBC1986 [32, 38].

The trajectory cluster *Type 2* adds another chapter to the discussion around the adiposity rebound point. Since it was first described [8], there has been discussion on what the AR reflects and why it occurs [39–42], despite the evidence found that the timing of AR predicts later life BMI and obesity [43–46]. The *Type 2* trajectories typically have a trough, instead of a clear nadir, following the rapid decrease in BMI directly after infancy peak, and before the BMI starts to rise towards the final state. Due to this trough, it is unclear how AR should be defined for these trajectories. Börnhorst et al. discussed that excluding children with inestimable infancy peak or AR may introduce selection bias and suggested using BMI at selected timepoints as a predictor for later BMI [47]. Based on the results presented here, we cannot warrant the maturational clusters’ predictability for later life BMI. However, we argue, that rather than ignoring the timing aspect and using BMI in one selected time point, it could be beneficial to study the use of maturational clusters presented here due to their longitudinal aspect.

Our sensitivity analysis using linear interpolation for height and weight showed 58,5% cluster agreement with our main analysis resulting in visually similar clusters. This may indicate that the BMI trajectories appearing in matching clusters form the “core” of the clusters, whereas those that change clusters are noisier and thus do not have a perfectly fitting group for them. Noise in this context may be induced by factors, such as nutrition, psychological stress or adverse health conditions, that can either have permanent or temporary effects on height or weight gain [48–52].

Although BMI as a measure of maturity has not been widely explored, few studies have recognised an inverse association between the age at AR and skeletal maturity [8, 53], and a positive association with age at menarche in girls [54]. As the childhood BMI is a dynamic trait that keeps evolving even after the AR, we again argue, that a longitudinal assessment in the timing could be more beneficial. Alternatively, a measure of phase distance could be used to differentiate longitudinal timing of different trajectories, where applicable.

In addition to the large sample size and long follow-up period, the Northern Finland Birth Cohort studies have relatively dense sampling and coverage improving the quality of height and weight modelling, which can be accounted as a strength of the study. The three trajectory clusters are a solid finding in our investigations. As well as being present in both sexes, similar clusters were also found in the sensitivity analysis, indicating that the pre-processing steps for obtaining height and weight estimates from exactly same time points did not steer the results of the clustering. Furthermore, we found similar clusters in females and males also when analysing the NFBC1966 and NFBC1986 separately in preliminary analysis.

We also acknowledge certain limitations to this study. Our inclusion criteria on the measurement density reduced the sample size substantially. The excluded sample had on average, 15 height and 16 weight observations per participant, but either their follow-up period was not long enough or had large gaps between consecutive measurements. In both cohorts, the density of height and weight observations were high especially in the first year but reduced after reaching school age. In addition to the reduction of sample size, poorly fitting height and weight trajectories were assessed manually, and decisions regarding model fitness were somewhat subjective. Although the data set is rich in growth measurements, information on lifestyle factors that could affect growth is mostly available around birth and adolescence missing out the mid-childhood.

There have been recent developments in characterising subtypes of obesity beyond BMI according to the pathophysiological mechanisms [55] and cardiometabolic risk profiles [56, 57]. Less work has been done to determine whether similar or comparable categories exist in children [58] and whether they could be explained by differential patterns of growth. We may speculate that the subtypes of adult obesity could start exhibiting differences in other factors (e.g. timing) as well as BMI levels in their early developmental trajectories.

We propose multiple steps to validate these results to ensure that the observed clustering, and possible new ones, could be translated into a better understanding of BMI maturation and ultimately more personalised clinical applications. To validate our results, the clustering should be replicated with other birth cohorts, ideally from diverse population samples considering among other factors ethnicities, geographical background and generations. Besides replication in other settings, more research is required to ascertain clinical relevance and implications to later life health outcomes associated with the different BMI patterns, as well as underlying causes of these differences. Furthermore, comparisons of different measures of biological maturation could provide insight and help validate the maturational properties of the identified clusters. Skeletal maturity (i.e., bone age) or percentage of attained adult height, for instance, would be meaningful at any age in contrast to measures that reflect pubertal stage [59].

The present findings highlight that possible mismatch between chronological age and maturational age should be given more attention in the literature. Furthermore, it is important to recognise that assuming only one BMI pattern might be insufficient for instance when modelling longitudinal BMI. In the clinical setting, our results suggest that caution should be taken when using the BMI as a sole measure of children’s weight status, especially around the extremes of early and late biological maturation. This adds to previous studies including a detailed study by Cunningham et al. showing that a large proportion (32%) of adolescents who lived with obesity were with overweight at the age of 5 years [60]. However, they also warned that a substantial part of the adolescents with obesity (7.9%) were of normal weight as 5-year-olds. This was also confirmed in other populations [61], including one of the cohorts studied in the present report [62], showing that the patterns of BMI through the life course are associated with the risk of type 2 diabetes.

## Conclusion

In addition to the more frequently represented changes in BMI where the first peak (adiposity peak in infancy) is reached at around 9 months of age followed by a valley by the age of 5-6 years in so-called adiposity rebound point, our method discriminated two additional patterns of growth in two generations of children born in Finland in 1966 and 1985-86. The proportion of children per growth pattern did not differ by sex or any early determinants used, but *Type 2* pattern was less represented in the population of children born in 1985-86 suggesting a possible generational difference between the cohorts. This approach could be adapted to other longitudinal phenomena, including different developmental processes, with time-dependent fluctuation to assess patterns in their timing. Furthermore, it could guide improved identification of BMI patterns that may influence long term health including obesity and its comorbidities in the clinical settings.

## Supplementary information

Additional Information

## Data Availability

NFBC data are available from the University of Oulu, Infrastructure for Population Studies. Permission to use the data can be applied for research purposes via an electronic material request portal. In the use of data, we follow the EU general data protection regulation (679/2016) and the Finnish Data Protection Act. Please, contact the NFBC project center (NFBCprojectcenter(at)oulu.fi) and visit the cohort website (www.oulu.fi/nfbc) for more information.

## References

[1] Marron JS, Ramsay JO, Sangalli LM, Srivastava A. Functional data analysis of amplitude and phase variation. Stat Sci. 2015, 30; 10.1214/15-STS524.

[2] Telesca D, Inoue LYT. Bayesian hierarchical curve registration. J Am Stat Assoc. 2008;103:328–39. 10.1198/016214507000001139.

[3] Sangalli LM, Secchi P, Vantini S, Vitelli V. K-mean alignment for curve clustering. Comput Stat Data Anal. 2010; 10.1016/j.csda.2009.12.008.

[4] Gervini D, Gasser T. Self-modelling warping functions. J R Stat Soc Ser B Stat Methodol 2004;66:959–71. 10.1111/j.1467-9868.2004.B5582.x.

[5] Carroll C, Müller H-G, Kneip A. Cross-component registration for multivariate functional data, with application to growth curves. Biometrics. 2021;77:839–51. 10.1111/biom.13340.

[6] Park J, Ahn J. Clustering multivariate functional data with phase variation. Biometrics. 2017;73:324–33. 10.1111/biom.12546. 27218696 10.1111/biom.12546

[7] Silverwood RJ, De Stavola BL, Cole TJ, Leon DA. BMI peak in infancy as a predictor for later BMI in the Uppsala Family Study. Int J Obes 2009;33:929–37. 10.1038/ijo.2009.108. 10.1038/ijo.2009.108 19564879

[8] Rolland-Cachera MF, Deheeger M, Bellisle F, Sempé M, Guilloud-Bataille M, Patois E. Adiposity rebound in children: a simple indicator for predicting obesity. Am J Clin Nutr 1984;39:129–35. 10.1093/ajcn/39.1.129. 6691287 10.1093/ajcn/39.1.129

[9] Roy SM, Chesi A, Mentch F, Xiao R, Chiavacci R, Mitchell JA, et al. Body Mass Index (BMI) trajectories in infancy differ by population ancestry and may presage disparities in early childhood obesity. J Clin Endocrinol Metab 2015;100:1551–60. 10.1210/jc.2014-4028. 25636051 10.1210/jc.2014-4028 PMC4399305

[10] Aris IM, Rifas-Shiman SL, Li L-J, Kleinman K, Coull BA, Gold DR, et al. Pre-, perinatal, and parental predictors of body mass index trajectory milestones. J Pediatr 2018;201:69–77. 10.1016/j.jpeds.2018.05.041. 29960766 10.1016/j.jpeds.2018.05.041 PMC6153023

[11] Aris IM, Bernard JY, Chen L-W, Tint MT, Pang WW, Lim WY, et al. Infant body mass index peak and early childhood cardio-metabolic risk markers in a multi-ethnic Asian birth cohort. Int J Epidemiol 2017;46:513–25. 10.1093/ije/dyw232. 27649801 10.1093/ije/dyw232 PMC5231275

[12] Camier A, Cissé AH, Lioret S, Bernard JY, Charles MA, Heude B, et al. Infant feeding practices associated with adiposity peak and rebound in the EDEN mother–child cohort. Int J Obes 2022;46:809–16. 10.1038/s41366-021-01059-y. 10.1038/s41366-021-01059-y 34980907

[13] Wen X, Kleinman K, Gillman MW, Rifas-Shiman SL, Taveras EM. Childhood body mass index trajectories: modeling, characterizing, pairwise correlations and socio-demographic predictors of trajectory characteristics. BMC Med Res Methodol 2012;12:38 10.1186/1471-2288-12-38. 22458308 10.1186/1471-2288-12-38 PMC3375197

[14] Eideh H, Jonsson B, Hochberg Z. Growth of the Kalahari Desert’s bushman - the Ju/’hoansi San. Acta Paediatr. 2012;101:528–32. 10.1111/j.1651-2227.2011.02573.x. 22181813 10.1111/j.1651-2227.2011.02573.x

[15] Gillison FB, Grey EB, Cumming SP, Sherar LB. Does adjusting for biological maturity when calculating child weight status improve the accuracy of predicting future health risk? BMC Public Health. 2021;21:1979 10.1186/s12889-021-12037-4. 34727900 10.1186/s12889-021-12037-4 PMC8561871

[16] Malina RM, Bouchard C, Bar-Or O. Growth, maturation, and physical activity. 2nd ed. Human Kinetics, Champaign, IL, 2004.

[17] University of Oulu. University of Oulu: Northern Finland Birth Cohort 1966. 1966. https://etsin.fairdata.fi/dataset/716939c3-7a2a-4b6a-91f3-92aca09bc52d.

[18] Nordström T, Miettunen J, Auvinen J, Ala-Mursula L, Keinänen-Kiukaanniemi S, Veijola J, et al. Cohort Profile: 46 years of follow-up of the Northern Finland Birth Cohort 1966 (NFBC1966). Int J Epidemiol 2022;50:1786–7. 10.1093/ije/dyab109. 34999878 10.1093/ije/dyab109 PMC8743124

[19] University of Oulu. University of Oulu: Northern Finland Birth Cohort 1986. 1986. https://etsin.fairdata.fi/dataset/f22c6599-2293-42bd-b65a-1a77945ed613.

[20] Järvelin M-R, Hartikainen-Sorri A-L, Rantakallio P. Labour induction policy in hospitals of different levels of specialisation. BJOG Int J Obstet Gynaecol 1993;100:310–5. 10.1111/j.1471-0528.1993.tb12971.x. 10.1111/j.1471-0528.1993.tb12971.x 8494831

[21] Järvelin M-R, Elliott P, Kleinschmidt I, Martuzzi M, Grundy C, Hartikainen A-L, et al. Ecological and individual predictors of birthweight in a northern Finland birth cohort 1986. Paediatr Perinat Epidemiol 1997;11:298–312. 10.1111/j.1365-3016.1997.tb00007.x. 9246691 10.1111/j.1365-3016.1997.tb00007.x

[22] Ramsay JO, Graves S, Hooker G. fda: Functional Data Analysis. 2021. https://CRAN.R-project.org/package=fda.

[23] Blu T, Thevenaz P, Unser M. Linear interpolation revitalized. IEEE Trans Image Process 2004;13:710–9. 10.1109/TIP.2004.826093. 15376602 10.1109/tip.2004.826093

[24] Tucker JD, Wu W, Srivastava A. Generative models for functional data using phase and amplitude separation. Comput Stat Data Anal 2013;61:50–66. 10.1016/j.csda.2012.12.001.

[25] Srivastava A, Wu W, Kurtek S, Klassen E, Marron JS. Registration of functional data using fisher-rao metric. ArXiv11033817. 2021; http://arxiv.org/abs/1103.3817.

[26] Tucker JD. fdasrvf: Elastic Functional Data Analysis. 2021. https://CRAN.R-project.org/package=fdasrvf.

[27] Rousseeuw PJ, Kauffman L. Wiley series in probability and statistics: an introduction to cluster analysis, finding groups in data: an introduction to cluster analysis. Wiley, New York, NY, 1990.

[28] Maechler, M, Rousseeuw, P, Struyf, A, Hubert, M and Hornik, K cluster: Cluster Analysis Basics and Extensions. 2022. https://CRAN.R-project.org/package=cluster.

[29] R Core Team. R: A Language and Environment for Statistical Computing. R Foundation for Statistical Computing, 2023. https://www.R-project.org/.

[30] Aris IM, Rifas-Shiman SL, Li L-J, Kleinman KP, Coull BA, Gold DR, et al. Patterns of body mass index milestones in early life and cardiometabolic risk in early adolescence. Int J Epidemiol 2019;48:157–67. 10.1093/ije/dyy286. 30624710 10.1093/ije/dyy286 PMC6380298

[31] Péneau S, Giudici KV, Gusto G, Goxe D, Lantieri O, Hercberg S, et al. Growth trajectories of body mass index during childhood: Associated Factors and Health Outcome at Adulthood. J Pediatr 2017;186:64–71. 10.1016/j.jpeds.2017.02.010. 28283258 10.1016/j.jpeds.2017.02.010

[32] Nedelec R, Miettunen J, Männikkö M, Järvelin M-R, Sebert S. Maternal and infant prediction of the child BMI trajectories; studies across two generations of Northern Finland birth cohorts. Int J Obes 2021;45:404–14. 10.1038/s41366-020-00695-0. 10.1038/s41366-020-00695-0 33041325

[33] Koning M, Hoekstra T, de Jong E, Visscher TLS, Seidell JC, Renders CM. Identifying developmental trajectories of body mass index in childhood using latent class growth (mixture) modelling: associations with dietary, sedentary and physical activity behaviors: a longitudinal study. BMC Public Health. 2016;16:1128 10.1186/s12889-016-3757-7. 27793201 10.1186/s12889-016-3757-7 PMC5086035

[34] Tao M-Y, Liu X, Chen Z-L, Yang M-N, Xu Y-J, He H, et al. Fetal overgrowth and weight trajectories during infancy and adiposity in early childhood. Pediatr Res 2024;95:1372–8. 10.1038/s41390-023-02991-7. 38200323 10.1038/s41390-023-02991-7

[35] Ward ZJ, Long MW, Resch SC, Giles CM, Cradock AL, Gortmaker SL. Simulation of growth trajectories of childhood obesity into adulthood. N Engl J Med 2017;377:2145–53. 10.1056/NEJMoa1703860. 29171811 10.1056/NEJMoa1703860 PMC9036858

[36] Simmonds M, Llewellyn A, Owen CG, Woolacott N. Predicting adult obesity from childhood obesity: a systematic review and meta-analysis. Obes Rev 2016. 10.1111/obr.12334. 26696565 10.1111/obr.12334

[37] Hartikainen-Sorri A-L, Rantakallio P, Sipilä P. Changes in prognosis of twin births over 20 years. Ann Med 1990;22:131–5. 10.3109/07853899009147255. 2361008 10.3109/07853899009147255

[38] Couto Alves A, De Silva NMG, Karhunen V, Sovio U, Das S, Taal HR, et al. Gwas on longitudinal growth traits reveals different genetic factors influencing infant, child, and adult BMI. Sci Adv 2019;5:3095 10.1126/sciadv.aaw3095. 10.1126/sciadv.aaw3095 PMC6904961 31840077

[39] Dietz WH. Critical periods in childhood for the development of obesity. Am J Clin Nutr 1994;59:955–9. 10.1093/ajcn/59.5.955. 8172099 10.1093/ajcn/59.5.955

[40] Cole T. Children grow and horses race: is the adiposity rebound a critical period for later obesity? BMC Pediatr. 2004;4:6 10.1186/1471-2431-4-6. 15113440 10.1186/1471-2431-4-6 PMC394330

[41] Rolland-Cachera MF, Cole TJ. Does the age at adiposity rebound reflect a critical period? Pediatr Obes. 2019, 14; 10.1111/ijpo.12467. 10.1111/ijpo.12467 30253063

[42] Aronoff JE, Ragin A, Wu C, Markl M, Schnell S, Shaibani A, et al. Why do humans undergo an adiposity rebound? Exploring links with the energetic costs of brain development in childhood using MRI-based 4D measures of total cerebral blood flow. Int J Obes 2022;46:1044–50. 10.1038/s41366-022-01065-8. 10.1038/s41366-022-01065-8 PMC9050592 35136192

[43] Whitaker RC, Pepe MS, Wright JA, Seidel KD, Dietz WH. Early adiposity rebound and the risk of adult obesity. Pediatrics. 1998;101:5 10.1542/peds.101.3.e5. 10.1542/peds.101.3.e5 9481024

[44] Rolland-Cachera MF, Deheeger M, Maillot M, Bellisle F. Early adiposity rebound: causes and consequences for obesity in children and adults. Int J Obes 2006;30:S11–S17. 10.1038/sj.ijo.0803514. 10.1038/sj.ijo.0803514 17133230

[45] Williams SM, Goulding A. Patterns of growth associated with the timing of adiposity rebound. Obesity. 2009;17:335–41. 10.1038/oby.2008.547. 19057527 10.1038/oby.2008.547

[46] Hughes AR, Sherriff A, Ness AR, Reilly JJ. Timing of adiposity rebound and adiposity in adolescence. Pediatrics. 2014;134:1354–61. 10.1542/peds.2014-1908. 10.1542/peds.2014-1908 25311600

[47] Börnhorst C, Siani A, Tornaritis M, Molnár D, Lissner L, Regber S, et al. Potential selection effects when estimating associations between the infancy peak or adiposity rebound and later body mass index in children. Int J Obes 2017;41:518–26. 10.1038/ijo.2016.218. 10.1038/ijo.2016.218 27899810

[48] Batista RFL, Silva AAM, Barbieri MA, Simões VMF, Bettiol H. Factors associated with height catch-up and catch-down growth among schoolchildren. PLoS ONE. 2012;7:32903 10.1371/journal.pone.0032903. 10.1371/journal.pone.0032903 PMC3299714 22427907

[49] Pervanidou P, Chrousos GP. Metabolic consequences of stress during childhood and adolescence. Metabolism. 2012;61:611–9. 10.1016/j.metabol.2011.10.005. 22146091 10.1016/j.metabol.2011.10.005

[50] Vanaelst B, Michels N, Clays E, Herrmann D, Huybrechts I, Sioen I, et al. The association between childhood stress and body composition, and the role of stress-related lifestyle factors—cross-sectional findings from the baseline ChiBS survey. Int J Behav Med 2014;21:292–301. 10.1007/s12529-013-9294-1. 23377786 10.1007/s12529-013-9294-1

[51] Keery H, LeMay-Russell S, Barnes TL, Eckhardt S, Peterson CB, Lesser J, et al. Attributes of children and adolescents with avoidant/restrictive food intake disorder. J Eat Disord 2019;7:31 10.1186/s40337-019-0261-3. 31528341 10.1186/s40337-019-0261-3 PMC6739995

[52] Auricchio R, Stellato P, Bruzzese D, Cielo D, Chiurazzi A, Galatola M, et al. Growth rate of coeliac children is compromised before the onset of the disease. Arch Dis Child 2020;105:964–8. 10.1136/archdischild-2019-317976. 32354718 10.1136/archdischild-2019-317976

[53] Williams S, Davie G, Lam F. Predicting BMI in young adults from childhood data using two approaches to modelling adiposity rebound. Int J Obes 1999;23:348–54. 10.1038/sj.ijo.0800824. 10.1038/sj.ijo.0800824 10340811

[54] Williams S, Dickson N. Early growth, menarche, and adiposity rebound. The Lancet. 2002; 10.1016/S0140-6736(02)07715-2. 10.1016/S0140-6736(02)07715-2 11867115

[55] Acosta A, Camilleri M, Abu Dayyeh B, Calderon G, Gonzalez D, McRae A, et al. Selection of antiobesity medications based on phenotypes enhances weight loss: a pragmatic trial in an obesity clinic. Obesity. 2021;29:662–71. 10.1002/oby.23120. 33759389 10.1002/oby.23120 PMC8168710

[56] Vecchié A, Dallegri F, Carbone F, Bonaventura A, Liberale L, Portincasa P, et al. Obesity phenotypes and their paradoxical association with cardiovascular diseases. Eur J Intern Med 2018;48:6–17. 10.1016/j.ejim.2017.10.020. 29100895 10.1016/j.ejim.2017.10.020

[57] Coral, DE, Smit, F, Farzaneh, A, Gieswinkel, A, Tajes, JF, Sparsø, T, et al. Subclassification of obesity for precision prediction of cardiometabolic diseases. Nat Med. 2024; 10.1038/s41591-024-03299-7. 10.1038/s41591-024-03299-7 PMC11835733 39448862

[58] Manco M, Helgason T, Körner A, Nowicka P, O’Malley G, Baker JL. Time for a new framework that treats obesity in children as an adiposity-based chronic disease. Nat Med 2024;30:3396 10.1038/s41591-024-03292-0. 39379707 10.1038/s41591-024-03292-0

[59] Beunen GP, Rogol AD, Malina RM. Indicators of biological maturation and secular changes in biological maturation. Food Nutr Bull 2006;27:244–56. 10.1177/15648265060274S508. 10.1177/15648265060274S508 17361661

[60] Cunningham SA, Kramer MR, Narayan KMV. Incidence of childhood obesity in the United States. N Engl J Med 2014;370:403–11. 10.1056/NEJMoa1309753. 24476431 10.1056/NEJMoa1309753 PMC4017620

[61] Bjerregaard LG, Jensen BW, Ängquist L, Osler M, Sørensen TIA, Baker JL. Change in overweight from childhood to early adulthood and risk of type 2 diabetes. N Engl J Med 2018;378:1302–12. 10.1056/NEJMoa1713231. 29617589 10.1056/NEJMoa1713231

[62] Bjerregaard LG, Wasenius N, Nedelec R, Gjærde LK, Ängquist L, Herzig K-H, et al. Possible modifiers of the association between change in weight status from child through adult ages and later risk of type 2 diabetes. Diab Care. 2020;43:1000–7. 10.2337/dc19-1726. 10.2337/dc19-1726 32139388

