# Supplementary material for: Timing based clustering of childhood BMI trajectories reveals differential maturational patterns; Study in the Northern Finland Birth Cohorts 1966 and 1986
Source: Int J Obes (Lond). 2025 Jan 16;49(5):872–80. doi: 10.1038/s41366-025-01714-8 (PMC12095082; doi:10.1038/s41366-025-01714-8)
Supplement: Supplementary file 1 — Additional Information [file 41366_2025_1714_MOESM1_ESM.pdf]

Additional Information

Heiskala, Tucker, Choudhary, Nedelec, Ronkainen, Sarala, Järvelin, Sillanpää and Sebert: "Timing based clustering of childhood BMI trajectories reveal differential maturational patterns; Study in the Northern Finland Birth Cohorts 1966 and 1986".

Contents

Additional Information 1

S1. Supplementary material and methods 3

S1.1 Classification of demographic variables 3

S1.2 B-spline interpolation 3

S1.3 Linear interpolation 4

S1.4 Aligning to cluster centroid and assessment of mean BMI level per cluster 4

S2. Supplementary results 5

S2.1 Attrition 5

S2.2 Clustering results when using linear interpolation to height and weight measurements 5

Supplementary Tables 6

Supplementary Table 1. Characteristics of the study sample and the excluded sample. With continuous variables mean (standard deviation, SD) and with categorical variables, n(%) reported. 7

Supplementary Table 2. Average silhouette widths in females and males for all numbers of cluster, *k* tested. 8

Supplementary Table 3. Average silhouette widths in females and males using linear interpolation for height and weight measurements. 9

Supplementary Table 4. Number of participants clustered to *Type 1, 2* and *3* in B-spline interpolation (main analysis) falling into corresponding clusters *Type A, B* and *C* in linear interpolation (sensitivity analysis). 10

Supplementary figures 11

Supplementary Figure 1. Description of steps to obtain BMI phase clusters from repeated height and weight measurements. 12

Supplementary Figure 2. Number of included participants at every inclusion criteria step. 13

Supplementary Figure 3. All trajectories within each cluster. The medoid trajectory is highlighted in black. Clusters of females are presented above and clusters of males below. 14

Supplementary Figure 4. Cluster specific trajectories of cross-sectional means and the corresponding 95% confidence intervals shaded. In each cluster, all curves were aligned to the medoid trajectory to allow meaningful representation on average BMI levels per cluster type. The time axis is transformed into *system time*, as a percentage of the total 16 years follow-up. 15

Supplementary Figure 5. Heatmaps of cluster-wise ordered pairwise phase distances in females on the left and males on the right. Height and weight measurements were processed using linear interpolation. Each row and column represent an individual. Each intersection point displays the phase distance

between the trajectories of the corresponding individuals as a colour. Blue colour indicates distance close to zero and yellow and red colours indicate greater distance. 16

Supplementary Figure 6. Medoid BMI trajectories in sensitivity analysis where height and weight measurements were fitted using linear interpolation. Females medoid trajectories are presented above and males below. 17

Supplementary Figure 7. Cluster specific trajectories from the sensitivity analysis where height and weight measurements were processed using linear interpolation. Medoid trajectories are highlighted in black. Clusters of females are presented above and males below. 18

References 19

Supplementary script 20

Fitting B-splines 20

Phase distances based on BMI 21

Clustering 22

Linear interpolation 22

S1. Supplementary material and methods

S1.1 Classification of demographic variables

Maternal cigarette smoking status was categorised into “no” if the mother had not smoked during pregnancy, “yes, quitted” if the mother gave up smoking by the end of second month of the pregnancy and “yes” if the mother continued smoking after the second month. Maternal educational attainment was reported during pregnancy, and the variable was labelled as “high” if there were additional studies after the national matriculation examination, “medium” if matriculation was finished without further studies, and “low” for all below matriculation [1]. Parity was categorised into “no” if the offspring was the mother’s first live born baby, and “yes” if it was not their first live birth. Mode of delivery was reported in three categories: non-instrumental vaginal delivery, caesarean section, and non-invasive assisted delivery [2]. Mother’s place of residence was self-reported of the time of birth, with three categories reflecting population density and availability of services: city or town, smaller village centre and remote village.

S1.2 B-spline interpolation

Height and weight were interpolated in one cohort at a time in order to derive BMI estimates in desired timepoints. Trajectories were fit one at a time with set parameters as described below.

To reach the spline fit over the first interpolation point for better behaviour around the edge value, birth length (or weight) was included in the analysis if the first height or weight measurement was recorded after one month of age. If there was a gap of 3.5 years between subsequent measurements, then a supporting point was added to the mean of the two observations.

We originally fitted cubic B-splines to individual height and weight data by cohorts as this degree is sometimes thought of as a standard degree in biomedical applications for its property of appearing “perfectly smooth to human eye” [3]. In NFBC1986, we were satisfied with the fit on degree of three. In NFBC1966 degree of three seemed too flexible and we decreased to quadratic B-splines. In all splines, knot points were placed at the individual’s minimum and maximum age as well as at 3, 12, 48, 96 and 144 months. Smoothing was applied on a lambda value of 1e-2 and penalising the 1st derivative on NFBC1966 and the 2nd derivative on NFBC1986. Height and weight were interpolated in every month from 1 month of age to 16 years. Degrees of best fitting splines within cohort were determined by visually inspecting randomly selected individual’s curves, as well as by searching for decreases (of 1 cm in height or 2 kg in weight) between the selected timepoints of 1, 4, 7, 10, 13 and 18 months and from there on at every 6 months until the age of 16 years. In the final fits, we assessed rapidly decreasing trajectories again, and excluded the participant from further analysis where the trajectory seemed to have spline fit anomalies. These anomalies meant either a sharp bend or hook shape between the observations or a decreasing tail to the spline that was not observable from the measurements.

B-spline interpolation was carried out using the R package `fda` [4] and an example script can be found in the section ‘Supplementary Script’. For a shorter introduction to B-splines with formal definition we refer readers to [5] and for a thorough introduction to [6].

S1.3 Linear interpolation

We used standard piecewise linear interpolation [7] for height and weight curves to assess whether our choice of harmonising them with cubic and quadratic base splines affected the clustering results. The selection criteria (1.-3.) for height and weight curves were the same as described in the main article for base splines. Furthermore, in linear interpolation we used birth length or birth weight if the first measurement was taken after the age of three months, as it is a requirement to have measures from the whole range estimated. Linear interpolation was conducted with the R function `approx` in the package `stats`.

After the interpolation step, we applied box filter [8] with five iterations for smoothing the curves. BMI estimates were calculated from the extracted height and weight estimates as described in the methods, and the same methodology was used for calculating the phase distances and clustering. Box filtering was performed through the R package `fdasrvf` [9].

We did not exclude any height or weight curves based on inadequate fit as with base splines, and therefore the total number of participants with BMI estimates was slightly higher, n=6,241.

S1.4 Aligning to cluster centroid and assessment of mean BMI level per cluster

In order to assess cross-sectional BMI levels in different trajectory clusters, we wanted to make sure that the cross-section is in the same *system time* and not in the same *chronological age* between the children. That is, we avoid comparing the BMI levels of children in different phases of their BMI development. As an example, in a mean BMI trajectory where phase was not controlled, the trajectory around adiposity peak would be less defined, i.e. wider and lower than it would be when trajectories were aligned. This problem has been illustrated and described in different settings for example by Marron and colleagues [10] and by James [11]. Furthermore, controlling phase could reduce the risk of mixing biologically more mature children with those with overweight or obesity.

For assessing average BMI levels between the clusters, each trajectory was aligned to the corresponding cluster centroid according to the clusters obtained in the main analysis where height and weight measurements were pre-processed using B-splines. After all trajectories were aligned, we calculated cross-sectional mean BMI at each age and visualised the results with their 95% confidence intervals (Supplementary Figure 4). The time axis is transformed into system time, as a percentage of the total 16 years follow-up. We remark, however, that in this case the 100% is not referring to “full maturation” but to the full follow-up time. “Full maturation” would mean that all participants have reached their adult BMI, which we do not have information on.

The aligning was done using the function `pair_align_functions` in the package `fdasrvf` [9].

S2. Supplementary results

S2.1 Attrition

Participants included in this study were more likely to be from the more recent cohort NFBC1986. They had on average eight more height and weight measurements available than the excluded participants, were more likely born full or post-term and to a nulliparous mother (Supplementary Table 1). Furthermore, town and village centre residents were more represented in the selected participants than in the excluded sample.

Amongst the participants taken to the subsequent analysis, height and weight were densely measured, with a mean (standard deviation, SD) of 22.6 (4.6) observations for height and 24.0 (5.1) observations for weight. After fitting B-splines to height and weight, we excluded 33 participants in NFBC1966 for inadequate height spline fitting and 6 for inadequate weight spline fitting. The corresponding figures in NFB1986 were 10 with height and 24 with weight.

S2.2 Clustering results when using linear interpolation to height and weight measurements

In the sensitivity analysis of pre-processing height and weight measurements using linear interpolation, we identified three clusters in both sexes. Although the heatmaps in Supplementary Figure 5 showed less clear separation for the two largest clusters in both sexes, the average silhouette method suggested number of clusters $k=3$ in both cases (Supplementary Table 3). Visual inspection of the trajectory shapes (Supplementary Figures 6 and 7) showed similar characteristic trajectories to those presented in the main analysis. These clusters were named as *Type A*, *Type B* and *Type C* according to the decreasing order in cluster sizes, as with the main analysis. Numbers of matching participants between the two interpolation methods are presented in Supplementary Table 4. Overall, the cluster agreement was 58% in females and 59% in males.

Supplementary Tables

Supplementary Table 1. Characteristics of the study sample and the excluded sample. With continuous variables mean (standard deviation, SD) and with categorical variables, n(%) reported.

	Included		Excluded	
Variable/levels	Available*	Missing**, n(%)	Available*	Missing**, n(%)
**Cohort**		-		-
NFBC1966	3064 (25.2)		9072 (74.8)	
NFBC1986	3098 (32.7)		6368 (67.3)	
**Average number of height measurements**	22.6 (4.6)	-	15.0 (7.7)	6697 (43.7)
**Average number of weight measurements**	24.0 (5.1)	-	16.1 (8.2)	6683 (43.6)
**Maternal age at birth, years**	27.8 (5.9)	11 (0.2)	27.8 (6.3)	108 (0.7)
**Maternal smoking during pregnancy**		74 (1.2)		337 (2.2)
no	4810 (79.0)		11492 (75.8)	
yes, quitted	302 (5.0)		741 (4.9)	
yes	976 (16.0)		2922 (19.3)	
**Maternal pre-pregnancy BMI, kg/m^2**	22.6 (3.2)	263 (4.3)	22.8 (3.4)	1118 (7.3)
**Mode of delivery**		1882 (30.5)		5290 (34.5)
non-instrumental vaginal delivery	3341 (78.1)		7954 (77.9)	
C-section	577 (13.5)		1311 (12.8)	
other (vacuum extraction, forceps)	362 (8.5)		944 (9.2)	
**Multiple birth**		-		59 (0.4)
singleton	6030 (97.9)		15018 (97.3)	
twin	130 (2.1)		418 (2.7)	
triplet	2 (0.0)		4 (0.0)	
**Parity**		14 (0.2)		110 (0.7)
no	2189 (35.6)		4952 (32.2)	
yes	3959 (64.4)		10437 (67.8)	
**Gestational age, weeks**	39.9 (1.7)	93 (1.5)	39.8 (2.2)	672 (4.4)
**Birth status**		93 (1.5)		672 (4.4)
term (≥37 weeks)	5800 (95.6)		13833 (93.3)	
preterm (<37 weeks)	269 (4.4)		989 (6.7)	
**Birth weight, grams**	3521 (521)	-	3474 (610)	60 (0.4)
**Place of residence at birth**		14 (0.2)		115 (0.8)
town	3670 (43.4)		5415 (35.2)	
village centre	2021 (32.9)		4524 (29.4)	
remote village	1457 (23.7)		5444 (35.4)	

* For categorical variables, the proportions are relative to only those who have a value. Hence, they are comparable to the proportions in Table 2 in the main.

**The proportions are relative to all participants included/excluded in the clustering analysis.

Supplementary Table 2. Average silhouette widths in females and males for all numbers of cluster, *k* tested.

	Average silhouette width	
k	Females	Males
2	0.19	0.21
3	0.20	0.20
4	0.15	0.15
5	0.14	0.14
6	0.11	0.11
7	0.11	0.12
8	0.10	0.11
9	0.09	0.09
10	0.08	0.08

Supplementary Table 3. Average silhouette widths in females and males using linear interpolation for height and weight measurements.

	Average silhouette width	
k	Females	Males
2	0.106	0.093
3	0.111	0.095
4	0.061	0.088
5	0.059	0.046
6	0.064	0.049
7	0.044	0.045
8	0.042	0.043
9	0.039	0.029
10	0.028	0.026

Supplementary Table 4. Number of participants clustered to *Type 1, 2* and *3* in B-spline interpolation (main analysis) falling into corresponding clusters *Type A, B* and *C* in linear interpolation (sensitivity analysis).

			Spline interpolation				
		Cluster	Type 1	Type 2	Type 3	NA	Total
Linear interpolation	Males	Type A	1195	504	149	25	1873
		Type B	325	399	99	8	831
		Type C	149	70	273	7	499
	Females	Type A	970	499	69	25	1563
		Type B	418	475	39	9	941
		Type C	178	54	297	5	534

Supplementary figures

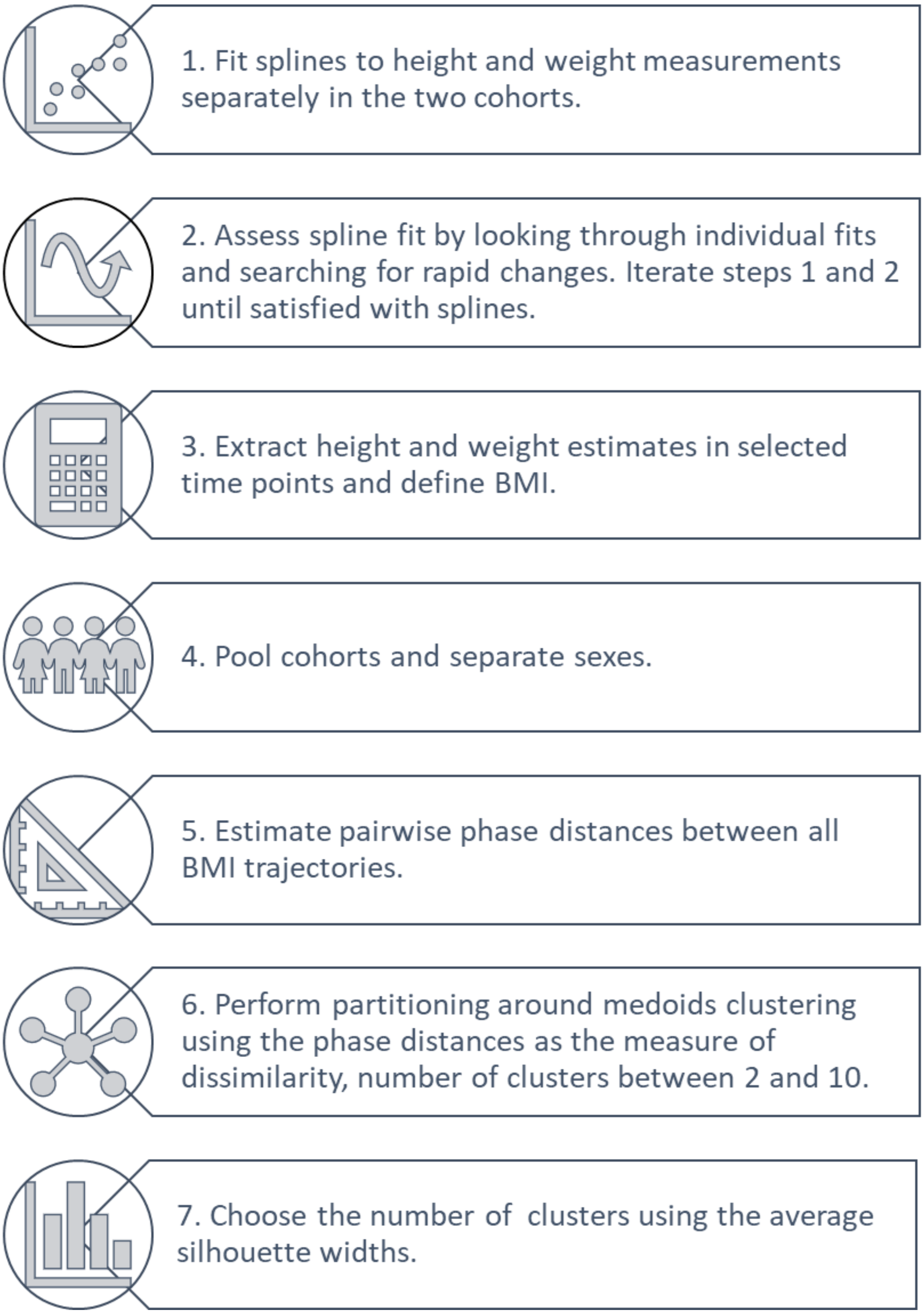

Supplementary Figure 1. Description of steps to obtain BMI phase clusters from repeated height and weight measurements.

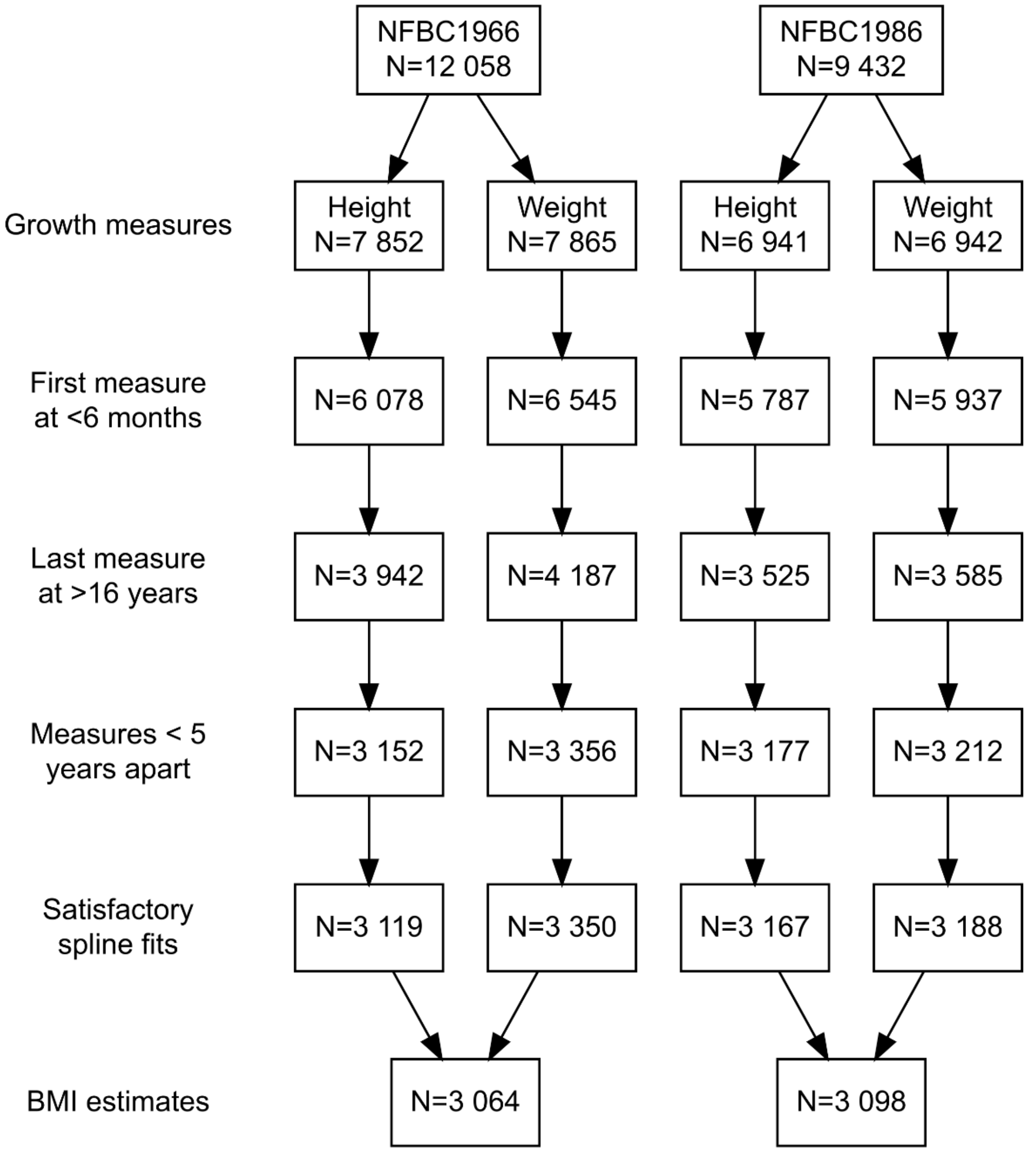

Supplementary Figure 2. Number of included participants at every inclusion criteria step.

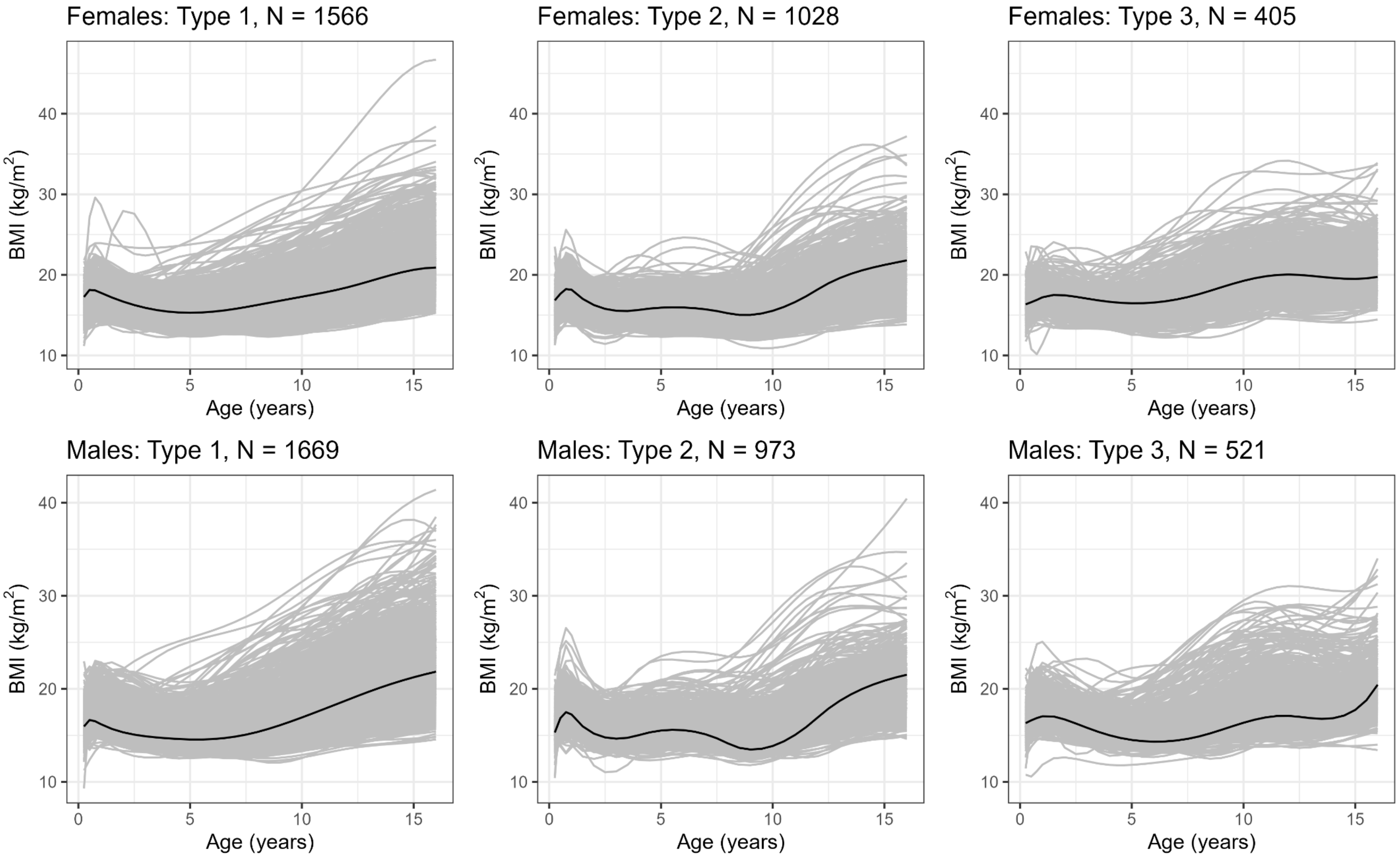

Supplementary Figure 3. All trajectories within each cluster. The medoid trajectory is highlighted in black. Clusters of females are presented above and clusters of males below.

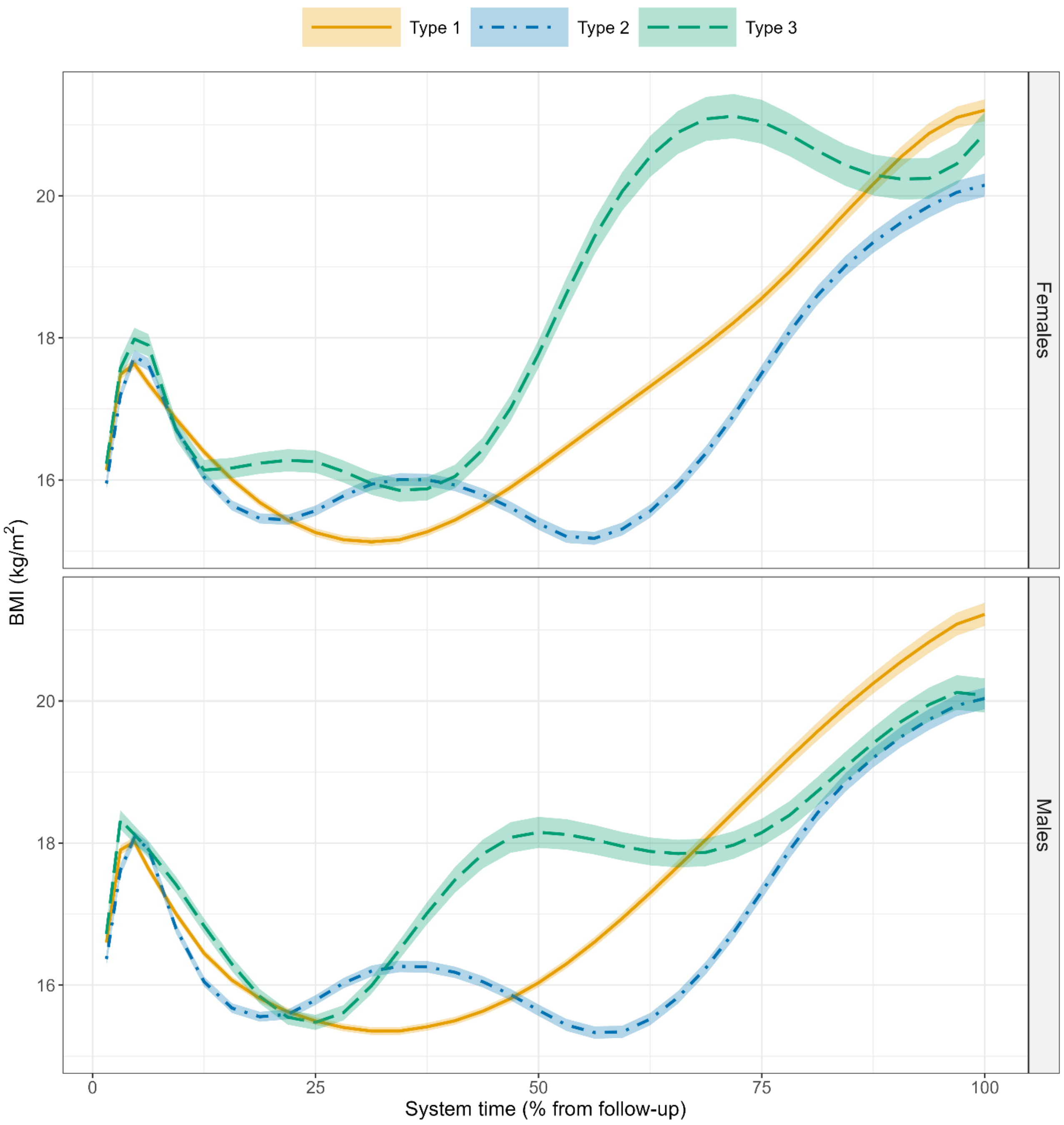

Supplementary Figure 4. Cluster specific trajectories of cross-sectional means and the corresponding 95% confidence intervals shaded. In each cluster, all curves were aligned to the medoid trajectory to allow meaningful representation on average BMI levels per cluster type. The time axis is transformed into *system time*, as a percentage of the total 16 years follow-up.

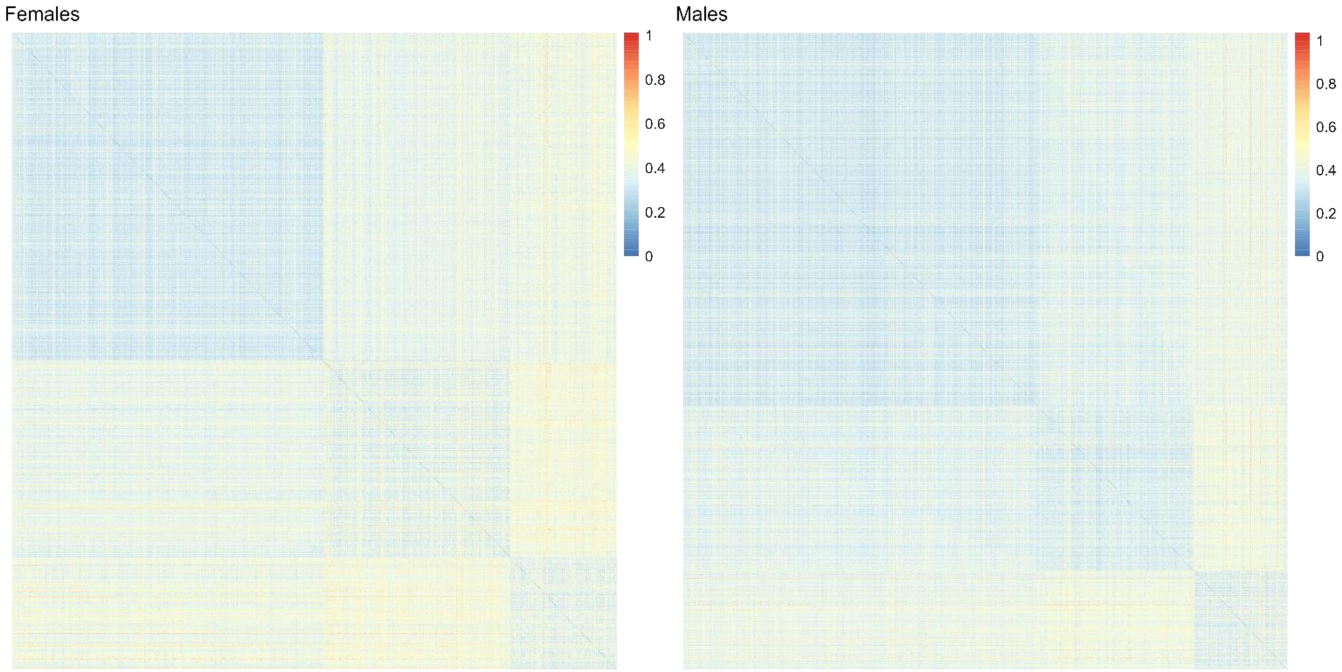

Supplementary Figure 5. Heatmaps of cluster-wise ordered pairwise phase distances in females on the left and males on the right. Height and weight measurements were processed using linear interpolation. Each row and column represent an individual. Each intersection point displays the phase distance between the trajectories of the corresponding individuals as a colour. Blue colour indicates distance close to zero and yellow and red colours indicate greater distance.

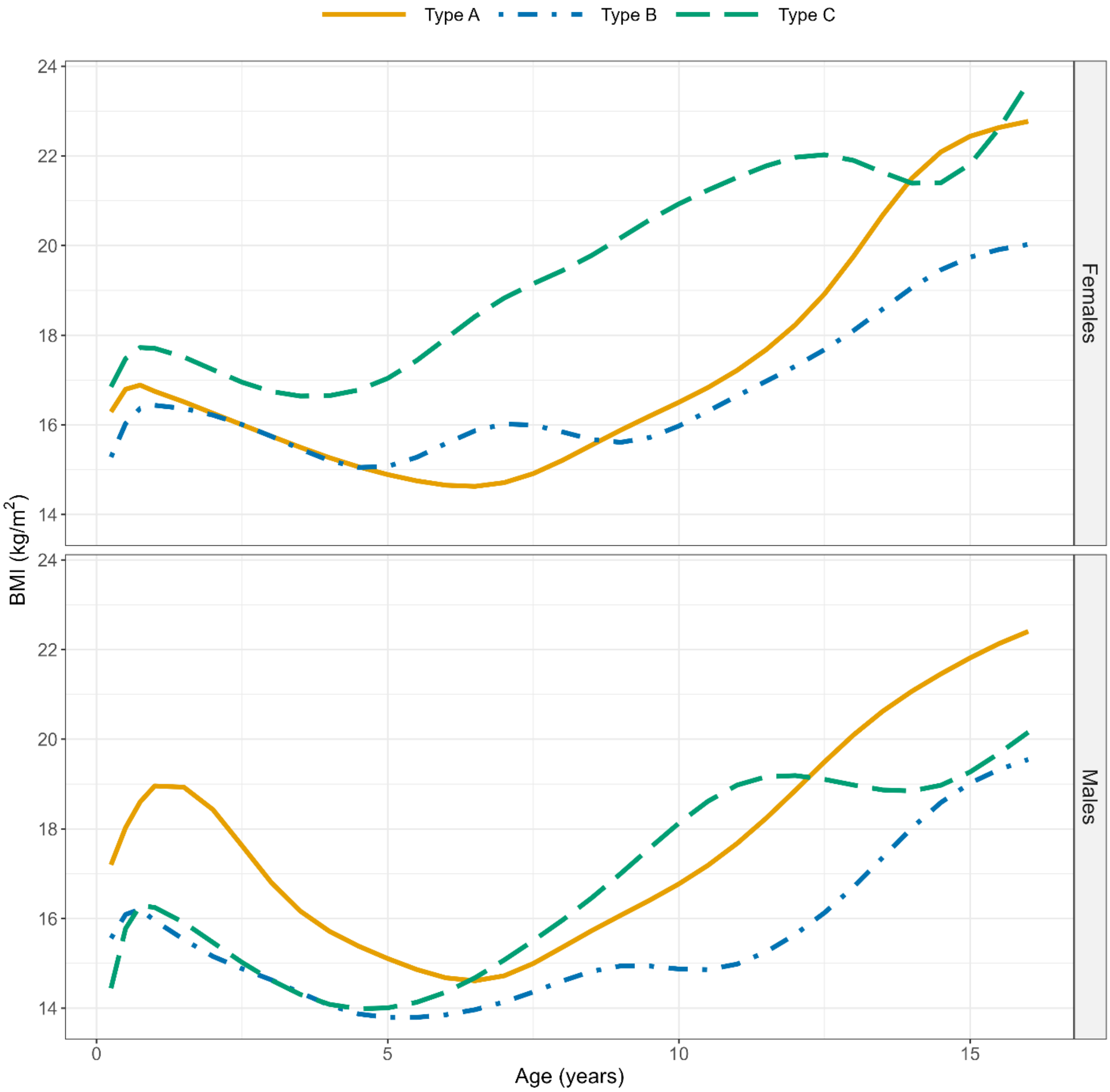

Supplementary Figure 6. Medoid BMI trajectories in sensitivity analysis where height and weight measurements were fitted using linear interpolation. Females medoid trajectories are presented above and males below.

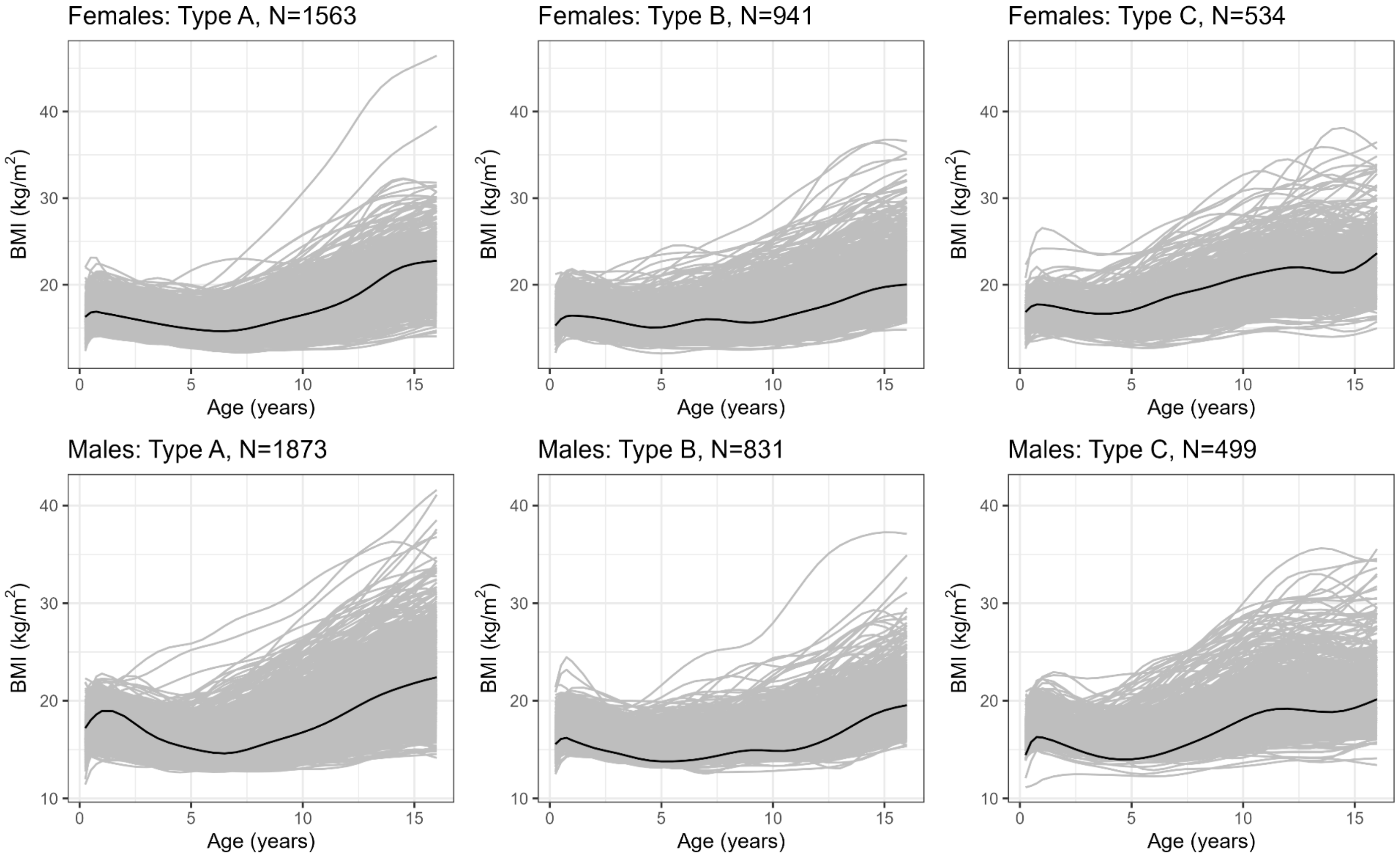

Supplementary Figure 7. Cluster specific trajectories from the sensitivity analysis where height and weight measurements were processed using linear interpolation. Medoid trajectories are highlighted in black. Clusters of females are presented above and males below.

References

[1] Cadman T, Elhakeem A, Vinther JL, et al. Associations of maternal education, area deprivation, proximity to greenspace during pregnancy and gestational diabetes with Body Mass Index from early childhood to early adulthood: A proof-of-concept federated analysis in seventeen birth cohorts. Epidemiology; 2022. https://doi.org/10.1101/2022.07.27.22278068.

[2] Nedelec R, Miettunen J, Männikkö M, Järvelin M-R, Sebert S. Maternal and infant prediction of the child BMI trajectories; studies across two generations of Northern Finland birth cohorts. Int J Obes. 2020:1–11. https://doi.org/10.1038/s41366-020-00695-0.

[3] Perperoglou A, Sauerbrei W, Abrahamowicz M, Schmid M. A review of spline function procedures in R. BMC Med Res Methodol. 2019;19:46. https://doi.org/10.1186/s12874-019-0666-3.

[4] Ramsay JO, Graves S, Hooker G. fda: Functional Data Analysis. 2021. https://CRAN.R-project.org/package=fda.

[5] Price MJ. Penalized b-splines and their application with an in depth look at the bivariate tensor product penalized b-spline. Ames, Iowa: Iowa State University; 2018. https://doi.org/10.31274/etd-180810-6071.

[6] Ramsay JO, Silverman BW. Functional data analysis. 2. edition. New York : Springer; 2005.

[7] Blu T, Thevenaz P, Unser M. Linear interpolation revitalized. IEEE Trans Image Process. 2004;13:710–719. https://doi.org/10.1109/TIP.2004.826093.

[8] Tucker JD, Wu W, Srivastava A. Generative models for functional data using phase and amplitude separation. Comput Stat Data Anal. 2013;61:50–66. https://doi.org/10.1016/j.csda.2012.12.001.

[9] Tucker JD. fdasrvf: Elastic Functional Data Analysis. 2021. https://CRAN.R-project.org/package=fdasrvf.

[10] Marron JS, Ramsay JO, Sangalli LM, Srivastava A. Functional Data Analysis of Amplitude and Phase Variation. Stat Sci. 2015;30:468–484. https://doi.org/10.1214/15-STS524.

[11] James GM. Curve alignment by moments. Ann Appl Stat. 2007;1:480–501. https://doi.org/10.1214/07-AOAS127.

Supplementary script

Fitting B-splines

Fit weight splines (adapted from the vignettes related to R package `fda`: https://www.psych.mcgill.ca/misc/fda/downloads/FDAfuns/R/demo/growthsmooth.R).

The script below fits B-splines for weight in NFBC1986.

```
library(fda)

# indata, weight measurements, contains the following columns:
# ID | personal identifier
# age | measurement age in months
# weight | measured weight in kilograms

# bw, birth weights, contains the following columns:
# ID | personal identifier
# age | age at birth (0 months)
# weight | birth weight

# initialise a dataset where the interpolated weights will be saved:
wgtout_nfbc1986=data.frame(ID =rep(in_ID,each=192),weight =as.numeric(NA))


for ( id in in_id){


    age = wt %>% filter(ID==id) %>% pull(age)
    wgt = wt %>% filter(ID==id) %>% pull(weight)

  # use birth height/weight if the first observation is later than 1 month
  if ( min(age) > 1){

    age0 = bw$age[which(bw$ID==id)]
    wt0 = bw$weight[which(bw$ID==id)]

    age = c(age0, age)
    wgt = c(wt0, wgt)
  }

  rng = c(min(age), max(age))
  knots = c(min(age), 3, 12, 48, 96, 144, max(age))
  norder   <- 3 # quadratic spline
  #norder = 4  #cubic spline
  nbasis = length(knots) + norder - 2
  wgtbasis <- create.bspline.basis(rng, nbasis, norder, knots)
  agefine_dt =  1:192

  Lfdobj    <- 1  # quadratic spline, penalise on 1st derivative
  #Lfdobj    <- 2 # cubic spline, penalise on 2nd derivative
  lambda    <- 1e-2
  growfdPar <- fdPar(wgtbasis, Lfdobj, lambda)
```

```
  wgtfd <- smooth.basis(age, wgt, growfdPar)$fd

  wgtout[wgtout$ID == id, "weight"] = eval.fd(agefine_dt, wgtfd)

}

wgtout_nfbc1986$age = rep(1:192,length(unique(wgtout_nfbc1986$ID)))
```

Look for sudden drops of 2kg or greater and visualise:

```
selected_age = c(1, 4, 7, 10, 13, seq(18, 192, by = 6) )
decr_2kg = character(0)
for (id in unique(wgtout_nfbc1986$ID)){
  wgt = round(wgtout_nfbc1986$weight[which(wgtout_nfbc1986$ID==id &
                                            wgtout$age %in% selected_age)],1)
  if (!all(diff(wgt) > - 2)) decr_2kg = c(decr_2kg, id)
}

pdf("WeightDecreaseOver2kg.pdf")
for (id in decr_2kg){
  # plot measurements with open circles
  plot(indata$age[indata$ID==id]*12, indata$weight[indata$ID==id],xlab="Age",
       ylab="Weight", main=id)
  # plot spline with line
  points(wgtout_nfbc1986$age[wgtout_nfbc1986$ID==id],
         wgtout_nfbc1986$weight[wgtout_nfbc1986$ID==id],
         type="l")
}
dev.off()
```

Decisions of exclusions based on these plots. Similar script is used for height and for both traits in NFBC1966. For calculating BMI the final set of time points is extracted.

Phase distances based on BMI

The script below is for calculating the phase distances for females (both cohorts pooled in the same data set). This step took ~3 hours with our data.

```
library(fdasrvf)
library(tidyverse)

# hw_F; height, weight and BMI estimates for females, contains
# ID | personal identifier
# age | age in years
# height | estimated height in cm
# weight | estimated weight in kg
# BMI | calculated based on height and weight

# extract ID's in separate vector
IDF = unique(hw_int_F$ID)

# hw_F data set into a matrix (N x M) of M functions (sets of BMI) with N sampl
es (IDs)
```

```
fun_F = hw_int_F %>% dplyr::filter(ID %in% IDF) %>% select(project_ID, BMI, age
)
fun_F = spread(fun_F, age, BMI)
fun_F_ID_order= fun_F$ID
fun_F = as.matrix(fun_F[, 2:ncol(fun_F)])
fun_F= t(fun_F)

time = hw_int_F$age[1:34]

# initialise empty distance matrix

F_distances = matrix(data = NA, nrow=length(IDF), ncol=length(IDF))

timestamp()
##############################
for(c in 1:(length(IDF)-1)){
  for(r in (c+1):length(IDF))
  F_distances[r, c] = elastic.distance(fun_F[,r],fun_F[,c], time)$Dx
}
##############################
timestamp()

colnames(F_distances) = fun_F_ID_order
rownames(F_distances) = fun_F_ID_order
```

Clustering

Exemplified with females. Uses `F_distances` from above.

```
library(cluster)

F_dist = as.dist(F_distances)

# partitioning around medoids clustering using the above defined phase distance
s:
pam_clusters = function(i) pam(F_dist, i, diss=TRUE)
clusters2to10 = lapply(2:10, pam_clusters)

# extract average silhouette widths into a dataframe for comparison:
 pam_2_10_sil_data =
   data.frame(k = 2:10, av_sil_width=unlist(lapply(clusters,
                                    function(x) x$silinfo$avg.width)))
```

Linear interpolation

The script below is for weight in NFBC1986.

```
library(tidyverse)

target_age = c(3,6,9,12,seq(18, 192, by = 6))

#initialise a data frame for the estimates
weight_dt = data.frame(ID = rep(in_id, each=length(target_age)),
                weight = as.numeric(NA),
```

```
                age = rep(target_age, length(in_id)))

  for ( id in in_id){

    age = wt %>% filter(ID==id) %>% pull(age)
    wgt = wt %>% filter(ID==id) %>% pull(weight)

  # use birth weight (birth lenght) if the earliest measure is after 3 months
    if ( min(age) > 3){

      age0 = bw$age[which(bw$ID==id)]
      wt_ext0 = bw$weight[which(d7$project_ID==id)]

      age = c(age0, age)
      wgt = c(wt_ext0, wgt)
    }


    dt[dt$project_ID == id, "weight"] =
      approx(age, wgt, xout = target_age, method="linear", ties="ordered")$y

  }

# reorganise data for the box filtering function
fun_wt = weight_dt %>% select(ID, weight, age)
fun_wt = spread(fun_wt, age, weight)
fun_wt_ID_order= fun_wt$ID
fun_wt = as.matrix(fun_wt[, 2:ncol(fun_wt)])
fun_wt= t(fun_wt)
# smooth using bx filter with 5 iterations:
library(fdasrvf)
smooth_wt = smooth.data(fun_wt, 5)
colnames(smooth_wt) = fun_wt_ID_order
smooth_weight=melt(smooth_wt, value.name = "weight")
smooth_weight = rename(age = Var1, project_ID = "Var2", smooth_weight)
```
